# Daily life and psychosocial functioning of adults with congenital heart disease: a 40–53 years after surgery follow-up study

**DOI:** 10.1007/s00392-022-02132-w

**Published:** 2022-12-19

**Authors:** C. Pelosi, R. M. Kauling, J. A. A. E. Cuypers, A. E. van den Bosch, W. A. Helbing, E. M. W. J. Utens, J. S. Legerstee, J. W. Roos-Hesselink

**Affiliations:** 1 Department of Cardiology, Erasmus Medical Center, Rotterdam, The Netherlands; 2 Department of Pediatrics, Division of Cardiology, Erasmus University Medical Center, Sophia Children’s Hospital, Rotterdam, The Netherlands; 3 Department of Child and Adolescent Psychiatry/Psychology, Erasmus Medical Center, Sophia Children’s Hospital, Rotterdam, The Netherlands; 4 Academic Center for Child Psychiatry Levvel/Amsterdam UMC, University of Amsterdam, Amsterdam, The Netherlands

**Keywords:** Congenital heart disease, Psychosocial functioning, Quality of life, Long-term follow-up

## Abstract

**Introduction:**

Nowadays, more than 90% of patients with congenital heart disease (CHD) reach adulthood. However, knowledge about their psychosocial functioning is limited.

**Methods:**

Longitudinal cohort study of patients (*n* = 204, mean age: 50 years, 46.1% female) who were operated during childhood (< 15 years) between 1968 and 1980 for one of the following diagnoses: atrial septal defect, ventricular septal defect, pulmonary stenosis, tetralogy of Fallot or transposition of the great arteries. Psychosocial functioning was measured every 10 years, using standardized and validated questionnaires. Results were compared with the general Dutch population and over time.

**Results:**

After a median follow-up of 45 [40–53] years adults with CHD had a significantly lower educational level, occupation level and employment rate, but better health-related quality of life and emotional functioning compared with normative data. Patients with moderate/severe defects reported significantly more self-perceived physical restrictions and lack of physical strength due to their CHD. Compared to 2011, in 2021 patients considered their CHD as more severe and they felt more often disadvantaged.

**Conclusions:**

Overall, despite a lower education, occupation level and employment rate, our sample of patients with CHD had a positive perception of their life and  their psychosocial functioning was even better than the norm. Although the quality of life was very good, their view on their disease was more pessimistic than 10 years ago, especially for patients with moderate/severe CHD.

**Graphic abstract:**

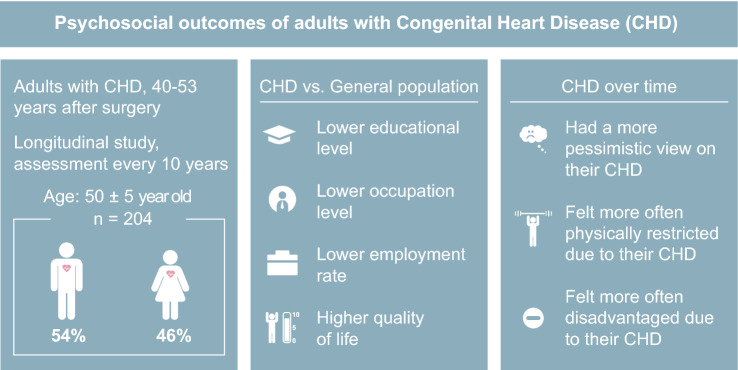

**Supplementary Information:**

The online version contains supplementary material available at 10.1007/s00392-022-02132-w.

## Introduction

In the last decades, major breakthroughs in diagnostic methods, surgical techniques and perioperative care have resulted in improved survival for children with congenital heart disease (CHD) with more than 90% of them reaching adulthood [1]. Therefore, focus has shifted to morbidity and more attention is warranted for the psychosocial functioning of this unique patient group. Consequently, the interest in psychological functioning and quality of life of adults born with CHD has increased. In fact, our previous studies in children and young adults with CHD showed difficulties in multiple psychosocial areas. Specifically, patients had a lower educational and occupational level [2–4]. However, little is known about the psychosocial outcomes of adults with a CHD when they reach their 40s and 50s. In this life stage, many patients are at risk of deterioration of their health after years of clinical stability, with fear for complications, such as heart failure and arrhythmias, and associated reduced life expectancy. Also, other acquired heart- and non-heart related diseases may occur. Furthermore, female patients can experience menopause, giving complaints which could be interpreted as cardiac symptoms (i.e., palpitations). All these factors could have a negative impact on the everyday life of patients with CHD, on their occupational or emotional functioning as well as on their quality of life.

A recent scientific statement of the American Heart Association emphasized that growing up with CHD may have a persistent social impact. In fact, these patients are more likely to face difficulties in social interaction as a result of their CHD [5]. Another study showed that 70% of patients with CHD experience uncertainties and worries about their medical condition  in the upcoming years [6].

To investigate long-term cardiological and psychosocial outcomes after surgery for CHD, the Erasmus Medical Center began a multidisciplinary longitudinal cohort study in 1991. This cohort was invited for extensive in-hospital investigations every ten years. The current report concerns the fourth follow-up of this same cohort of patients, now up to 50 years after surgery [3, 4, 7–14]. The aims of the present study were: (1) to compare the biographical characteristics and psychosocial functioning of adults with CHD with the general population; (2) to make comparisons between the diagnostic groups (mild vs moderate/severe) in terms of psychopathology [15]; and (3) to assess the changes of biographical characteristics and emotional and social functioning over time [2].

## Methods

### Inclusion criteria and patient sample

Patients diagnosed with atrial septal defects (ASD), ventricular septal defect (VSD), pulmonary stenosis (PS), tetralogy of Fallot (ToF) and transposition of the great arteries (TGA), who underwent their first heart surgery at Erasmus Medical Center between 1968 and 1980, and who were younger than 15 at the time of surgery, were included in the study. The previous follow-ups of the same cohort took place in 1991, 2001 and 2011 [6–12]. All patients diagnosed with TGA underwent Mustard procedure.

According to the classification of the European Society of Cardiology for adults with CHD, patients were classified as mild (ASD, VSD and PS) and moderate/severe CHD (ToF and TGA) [15].

We invited all 343 patients who were alive and participated in at least two of the previous follow-ups. Of this group of patients, 204 agreed to participate and completed the questionnaires. There was no difference in term of age, diagnosis and gender between participants (*n* = 204) and non-participants (*n* = 139).

### Assessment procedure

The local ethical committee approved the research protocol a priori (MEC-2019 0465) as complying to the ethical guidelines of the Declaration of Helsinki of 1975.

All the eligible patients were invited uniformly and provided written informed consent. The questionnaires were completed digitally on a secure website (GemsTracker, Copyright©2011,ErasmusMC and Equip Healthcare companies) before their visit to the hospital. All of them were invited to visit our outpatient clinic for extensive cardiological examinations. Due to technical issues or to personal patients’ preference, 46 patients completed the questionnaires on paper.

### Instruments

*Biographical characteristics*, i.e., living conditions, marital status, off-spring, educational level, working conditions and view on the CHD, were assessed through a questionnaire [2, 3].

Gender and age-specific normative data were derived from the general Dutch population from the Netherlands Bureau of Statistics (CBS) [16].

*Social functioning* The Perceived Social Support Scale (PSSS12) was used to assess the experience of direct social support in the patients′ personal environment. The PSSS12 is a 12-item questionnaire. The scoring system is based on a Likert- scale ranging from 1 (very strongly disagree) to 7 (very strongly agree), with a minimum total score of 12 and a maximum of 84. Three sub-scores can be calculated for family, friends and significant others. A high total score on the PSSS12 suggests a high level of social support [17]. No normative data were available.

*Psychosocial functioning* was assessed with the Dutch Personality Questionnaire (DPQ) [18]. The questionnaire contains 140 not overlapping items, divided in 7 scales. It has a response format of ‘yes = 2,’ ‘I don’t know’ = 1 and ‘no = 0.’ Only 4 scales of this questionnaire were used: neuroticism (feeling of stress, unstableness, insecurity and depression), social inadequacy (timid and close attitude), hostility (intolerance toward other people, extend of criticism and distrust) and self-esteem (energy, self-control, flexibility and positive attitude towards work). Higher scores on the self-esteem scale indicate higher self-esteem, whereas higher scores on the other scales are representative of poorer social adjustment. Normative data of the Dutch population are derived from the manual [18].

*Quality of Life* was assessed with the Satisfaction with Life Scale (SWLS) [19, 20]. This scale consists of a list of 5 items concerning satisfaction with life. Each statement is scored on a Likert- scale from 1 (strongly disagree) to 7 (strongly agree). An overall score is derived from the sum of all the individual items with a minimum score of 5 to a maximum of 35. Higher scores define better outcomes. This scale was proved to be a valid psychometric measure tool for patients with CHD [21]. Normative data were derived from the general Dutch population [19].

The Covid-19 index score of the day of completion of the questionnaire was selected per patient. This score measures, as a mean on 9 metrics (each scored from 0 to 100, strictest), the strictness of the governmental policies during the Covid-19 pandemic for each day since the beginning of it [22, 23].

### Statistical analyses

Continuous data are presented as mean ± standard deviation or median [25th–75th percentile] when appropriate. Normality was assessed using histograms and the Shapiro–Wilk test. Categorical data are presented as frequencies and percentages. Differences between continuous data were analyzed with independent *t*-test or Mann–Whitney *U* test. Continuous data were compared with normative data using the one sample *t*-test, while categorical variables were compared using the Chi-square test. Longitudinal analyses between 2011 and 2021 were performed with paired *t-*test for continuous paired data, whereas McNemar test or Stuart–Maxwell test was used for comparison between paired continuous data.

Univariable linear regressions (in supplementary material) were performed to identify possible biographical and medical predictors of quality of life (SWLS) and psychosocial functioning (DPQ) subscales. The following predictors were tested separately: age, gender, cardiac medications, Covid-19 stringency index, exercise capacity, systemic ventricular function, cardiac diagnosis, living condition, marital status, education and occupation level. Predictors with *p*-values < 0.20 were entered into a multivariable linear regression model (backward elimination; p < 0.05).

The statistic tests were two-sided and a *p* < 0.05 was considered significant. All the statistical analyses were performed using IBM Statistics SPSS v28.0 version for Windows and R software v. 4.2.1 for Windows.

## Results

### Biographical characteristics

The biographical characteristics of our cohort are described in Table 1.

**Table 1:** Biographical characteristics

	2021 vs norm			Congenital heart disease classification			Longitudinal development (*n* = 177)			
	Total 2021 (*n* = 204)	Norm (*n* = ~)	*p*	Mild	Moderate/ severe	*p*	2001	2011	2021	*P*^$^
*Biographical status*										
Female	46.1% (94)	–	–	48.2% (68)	41.3% (23)	0.357	–	–	–	–
Age (years)	50.0 ± 5.1	–	–	50.8 ± 5.1	48.3 ± 4.8	0.001	30.4 ± 5.1	40.4 ± 5.2	50.0 ± 5.1	–
*Systemic function*						< 0.001				0.061
Good	65.7% (117)	–	–	81.1% (103)	27.5% (14)		84.1% (132)	66.1% (82)	67.3% (105)	
Reasonable	23.0% (41)	–	–	15.7% (20)	41.2% (21)		7.6% (12)	21.8% (27)	21.2% (33)	
Moderate	9.6% (17)	–	–	3.1% (4)	25.5% (13)		6.4% (10)	10.5% (13)	9.6% (15)	
Bad	1.7% (3)	–	–	–	5.9% (3)		1.9% (3)	1.6% (2)	1.9% (3)	
*Cardiac medications (total)*	60.4% (113)	–	–	32.6% (43)	56.4% (31)	0.002	4.8% (7)	22.2% (39)	39.1% (63)	< 0.001
Beta-blockers	12.7% (26)	–	–	10.6% (15)	17.5% (11)	0.177	1.1% (2)	4.0% (7)	11.9%(21)	0.002
Ca-antagonists	6.4% (13)	–	–	6.4% (9)	6.3% (4)	0.993	–	1.1% (2)	6.8% (12)	0.002
Oral anticoagulant	17.6% (36)	–	–	14.9% (21)	23.8% (15)	0.123	0.6% (1)	4.0% (7)	16.4% (29)	< 0.001
Cholesterol lowering	10.8% (22)	–	–	9.9% (14)	12.7% (8)	0.556	–	4.0% (7)	11.3% (20)	< 0.001
Anti-hypertensive	19.6% (40)	–	–	12.1% (17)	36.5% (23)	< 0.001	2.3% (4)	6.8% (12)	18.1% (32)	< 0.001
Anti-arrhythmia	7.8%(16)	–	–	2.1% (3)	20.6% (13)	< 0.001	1.1% (2)	2.8% (5)	8.5% (15)	0.031
Others	9.8% (20)	–	–	8.5% (12)	12.7% (8)	0.353	1.1% (2)	6.2% (11)	7.3% (13)	0.815
Heart related hospitalizations^&^	28.6% (58)	–	–	20.0% (28)	47.6% (30)	< 0.001	10.9% (19)	26.1% (31)	27.7% (49)	0.124
Diabetes	4.5% (8)	–	–	3.0% (4)	7.0% (4)	0.203	–	–	–	–
Smokers	12.3% (25)	21.1%	0.09	13.6% (19)	9.5% (6)	0.417	–	13.7% (24)	13.6% (24)	1.0
Hypercholesterolemia	8.4% (16)	–	–	7.4% (10)	10.7% (6)	0.654	–	–	–	–
CVA/TIA^&&^	4.9% (10)	–	–	4.3% (6)	6.5% (4)	0.515	–	–	–	–
Exercise capacity (%)	96.7 ± 23.0	–	–	99.9 ± 22.9	89.1 ± 21.4	0.003	93.4 ± 19.4	94.3 ± 18.2	98.4 ± 24.2	0.001
*Living condition*			0.817			0.257				0.057
With parents	1.5% (3)	1.0%		2.1% (3)	–		12.5% (22)	4.5% (8)	1.1% (2)	
Independently	96.1% (196)	96.8%		95.7% (135)	96.8% (61)		86.9% (153)	95.5% (168)	97.2% (172)	
Institution	1.0% (2)	1.2%		1.4% (2)	3.2% (2)		–	–	0.6% (1)	
Other	1.5% (3)	1.0%		0.7% (1)	–		0.6% (1)	–	–	
*Marital status*			0.245			0.235				0.105
No (stable) relationship*	28.4% (58)	29.2%		27.7% (39)	30.2% (19)		45.5% (80)	27.3% (48)	25.4% (45)	
Married	58.3% (119)	56.1%		56.0% (79)	63.5% (40)		51.7% (91)	65.3% (115)	61.6% (109)	
Divorced	11.3% (23)	13.8%		13.5% (19)	6.3% (4)		2.8% (5)	6.8% (12)	10.7% (19)	
Widowed**	2.0% (4)	0.9%		2.8% (4)	–		–	0.6% (1)	2.3% (4)	
*Off-spring*			-			0.990				< 0.001
No	22.3% (45)	–		22.3% (31)	22.2% (14)		59.7% (105)	28.7% (50)	21.1% (37)	
≥ 1 child	77.7% (157)	–		77.7% (108)	77.8% (49)		40.3% (71)	71.3%(124)	78.9% (138)	
*Daily activity*^			< 0.001			0.426				0.012
Not working	10.6%(20)	3.1%		8.6% (12)	11.1% (7)		11.4% (20)	12.5% (22)	9.4% (19)	
Working	89.4%(168)	96.9%		85.0% (119)	77.8% (49)		86.4% (152)	86.4% (152)	82.8% (168)	
Unable to work	–	–		6.4% (9)	11.1% (7)		2.3% (4)	1.1% (2)	7.9% (16)	
*Educational level*^^			< 0.001			0.963				0.368
Low	33.8% (69)	17.2%		33.3% (47)	34.9% (22)		39.0%(67)	33.1% (58)	34.5% (61)	
Secondary	31.4% (64)	39.4%		31.9% (45)	30.2% (19)		37.8%(65)	32.6% (57)	31.1% (55)	
High	34.8% (71)	43.3%		34.8% (49)	34.9% (22)		23.2% (40)	34.3% (60)	34.5% (61)	

Normative data were derived from the Dutch Central Bureau of Statistics in 2021 and are based on the Dutch population aged 45–55 years old

*CVA* = cerebrovascular accident; *TIA* = transient ischemic attack

^$^*p*-value calculated on the differences between 2011 and 2021 on the 177 consecutive patients

^&^Reported heart-related hospitalizations in the last 10 years. It includes every hospitalization related to the heart problems such as for re-intervention, arrhythmias, coronary heart disease, endocarditis, pacemaker or ICD change, hypertension and heart failure

^&&^ Since surgery until 2021

^*^All patients with or without a (stable) relationship (11.8%) or that are living together without marriage (16.8%)

^**^All patients that have been widowed, regardless of their current status

*^The comparison with the norm data was done on the 188 patients able to work for conformity with the cbs data. "Not able to work" includes: disabled (n* = *13), long-term sick leave (n* = *1) and patient with work for mentally disabled (n* = *2)*

^^ Based on SOI (Standaard Onderwijsindeling (Standard Educational Divisions)) 2021 classification

Forty to 53 years after surgery, CHD patients still showed a significantly lower educational level than the reference group (33.8% vs 17.2%, *p* < 0.001). No difference in terms of educational level was found between mild and moderate/severe CHD.

Patients with mild CHD were operated at older age and consequently they were older at follow-up (50.8 ± 5.1 vs 48.3 ± 4.8, *p* = 0.001). The majority had good ventricular systemic function (81.1% vs 27.5%, *p* < 0.001), required less cardiac medications (32.6% vs 56.4%, *p* = 0.002) and had better exercise capacity (99.9 ± 22.9 vs 89.1 ± 21.4, *p* = 0.003) compared to their counterparts with moderate/severe CHD.

Patients with mild CHD were less often hospitalized for cardiac disease compared to those with moderate/severe CHD (20.0% vs 47.6%, *p* < 0.001).

Compared to 2011, we found that in 2021 patients had a better exercise capacity (98.4 ± 24.2 vs 94.3 ± 18.2, *p* = 0.001); however, more of them took cardiac medication (39.1% vs 22.2%, *p* < 0.001). In addition, in 2021 more patients had children (78.9% vs 71.3%, *p* < 0.001).

### Work and working conditions

In 2021, the main daily activity has significantly changed (less CHD patients were working (82.8% in 2021 vs 86.4% in 2011, *p* = 0.001) and more of them were unable to work (7.9% vs 1.1%, *p* = 0.012 for all categories).

Table 2 shows the results regarding work and working condition of our cohort. Compared to the normal Dutch population of the same age and gender, patients with CHD had significantly more often a low occupational level (*p* < 0,001 for all categories). Specifically, they were underrepresented in higher occupations (28.5% vs 38.5% of the general population). The 25.6% of adults with CHD had an average occupational level, against 19.8% in the general Dutch population. No significant difference was found in terms of work and working conditions between mild and moderate/severe CHD. Over time, no significant change was found in occupation level and working conditions. However, in 2021 more patients had an annual income higher than 36'000 euro's than in the previous follow-up (55.6% vs 32.0%, *p* < 0.001).

**Table 2:** Work and working conditions

	*2021 vs norm*			Congenital heart disease classification			Longitudinal development (*n* = 177)			
	Total 2021 (*n* = 204)	Norm (*n* = ~)	*p*	Mild(*n* = 135)	Moderate/ severe (*n* = 63)	*p*	2001	2011	2021	p*
Occupational level**			< 0.001			0.628				0.061
Elementary	9.3% (16)	5.5%		7.6% (9)	13.2% (7)		9.4% (15)	7.9% (13)	8.6% (13)	
Lower	36.6% (63)	36.2%		37.8% (45)	34.0% (18)		51.6% (82)	47.0% (77)	40.1% (61)	
Average	25.6% (44)	19.8%		26.9% (32)	22.6% (12)		21.4% (34)	25.6% (42)	25.0% (38)	
Higher	28.5% (49)	38.5%		27.7% (33)	30.2% (16)		17.6% (28)	19.5% (32)	26.3% (40)	
*Income*			0.797			0.517				< 0.001
< 36′000	42.7% (64)	45.40%		41.0% (43)	46.7% (21)		86.4% (108)	68.0% (102)	44.4% (59)	
≥ 36′000	57.3% (86)	54.50%		59.0% (62)	53.3% (45)		13.6% (17)	32.0% (48)	55.6% (74)	
*Sick leave compared to colleagues*						0.396				0.059
More	9.5% (17)	–	–	10.7% (13)	7.0% (4)		8.2% (12)	6.3% (9)	8.9% (14)	
Same	24.0% (43)	–	–	21.3% (26)	29.8% (17)		32.7% (48)	40.8% (58)	24.8% (39)	
Less	66.5% (119)	–	–	68.0% (83)	63.2% (36)		59.2% (87)	52.8% (75)	66.2% (104)	
Amount of hours work per week:***			0.234			0.528				0.134
Part-time	41.7% (73)	48.1%		40.2% (49)	45.3% (24)		28.4% (44)	37.3% (60)	42.6% (66)	
Full-time	58.3% (102)	51.8%		59.8% (73)	54.7% (29)		71.6% (11)	66.7% (32)	57.4% (89)	
*Reason part-time:*						0.226				0.165
Only heart	9.8% (5)	–	–	6.1% (2)	16.7% (3)		4.5% (2)	3.1% (2)	9.1% (5)	
Heart plays a role	5.9% (3)	–	–	3.0% (1)	11.1% (2)		4.5% (2)	10.8% (7)	3.6% (2)	
Not heart related	84.3% (43)	–	–	90.9% (30)	72.2% (13)		90.9% (40)	86.2% (56)	87.3% (48)	
*Limitation in choosing a profession because of CHD:*						0.133				0.596
A lot	5.4% (11)	–	–	4.3% (6)	7.9% (5)		–	5.7% (10)	5.7% (10)	
A little	9.4% (19)	–	–	7.1% (10)	14.3% (9)		–	6.8% (12)	8.0% (14)	
No	85.2% (173)	–	–	88.6% (124)	77.8% (49)		–	87.5% (154)	86.4% (152)	
*Perception of career opportunities:*						0.083				1.0
Same as colleagues	91.6% (163)	–	–	94.2% (114)	86.0% (49)		–	92.0% (127)	92.3% (144)	
Less than colleagues	8.4% (15)	–	–	5.8% (7)	14.0% (8)		–	8.0% (11)	7.7% (12)	
*Do you do physical work?*						0.423				0.086
Yes, always	13.3% (24)	–	–	14.5% (18)	10.7% (6)		–	12.7% (21)	14.5% (23)	
Yes, sometimes	24.4% (44)	–	–	21.8% (27)	30.4% (17)	–	–	16.9% (28)	23.9% (38)	
No	62.2% (112)	–	–	63.7% (79)	58.9% (33)	-	–	69.9% (116)	61.6% (98)	
I don't know	–	–	–	–	–	–	–	0.6% (1)	–	

Normative data were derived from the Dutch Central Bureau of Statistics in 2021 and are based on the Dutch population aged 45–55 years old

^*^p-value reflects the statistical significance of the differences between 2011 and 2021 of 177 consecutive patients

^**^Based on the ISCO 08 classification [41]

^***^Only patients who have a job of at least 12 h per week

### View on CHD

Compared to patients with mild CHD, patients with moderate/severe CHD experienced their heart condition as more severe (*p* < 0.001 for all categories). They felt more often physically restricted (*p* = 0.005 for all categories) and at disadvantage because of their CHD (*p* < 0.001, for all categories). Compared to 2011, in 2021 significantly more adults with CHD defined their condition as moderately severe (*p* < 0.001 for all categories), they felt more frequently physically restricted (*p* = 0.042, for all categories), they perceived significantly less physical strength (*p* < 0.001, for all categories) and they felt more often at disadvantage (*p* < 0.001, for all categories) because of their CHD. Furthermore, in 2021, patients felt significantly more restricted by their scars than in 2011 (*p* < 0.001 for all categories) (Table 3).

**Table 3:** View on CHD

	*2021*	Congenital heart disease classification			*Longitudinal development (n* = *177)*			
	Total 2021 (*n* = 204)	Mild (n-141)	Moderate/severe (*n* = 63)	p	2001	2011	2021	p*
Contact with other CHD patients	10.3% (21)	10.7% (15)	9.5% (6)	0.797	14.2% (25)	17.7% (31)	10.2% (18)	0.026
Visit psychological support in the last 10 years**	19.6% (40)	19.9% (28)	19.0% (12)	0.893	–	20.9%(37)	16.5% (29)	0.312
*How serious is your CHD*				< 0.001				< 0.001
Not serious	63.1% (128)	72.9% (102)	41.3% (26)		87.4% (153)	81.3% (143)	64.8% (114)	
Moderately serious	35.5% (72)	25.7% (36)	57.1% (36)		10.9% (19)	18.2% (32)	34.1% (60)	
Very serious/bad	1.5% (3)	1.4% (2)	1.6% (1)		1.7% (3)	0.6% (1)	1.1% (2)	
*Restriction due to scar*				0.736				< 0.001
Never	65.7% (134)	66.7% (94)	63.5% (40)		69.9% (123)	84.7% (149)	66.7% (118)	
Sometimes	28.4% (58)	27.0% (38)	31.7% (20)		23.3% (41)	9.7% (17)	28.2% (50)	
Often	5.9% (12)	6.4% (9)	4.8% (3)		6.8% (12)	5.7% (10)	5.1% (9)	
*Self-perceived physical restriction because of CHD*				0.005				0.042
Never	69.1% (141)	75.9% (107)	54.0% (34)		79.0% (139)	80.1% (141)	70.6% (125)	
Sometimes	19.6% (40)	16.3% (23)	27.0% (17)		15.9% (28)	14.2% (25)	19.8% (35)	
Often	11.3% (23)	7.8% (11)	19.0% (12)		5.1% (9)	5.7% (10)	9.6% (17)	
*Lack of physical strength because CHD*				0.210				< 0.001
Never	73.0% (149)	76.6% (108)	65.1% (41)		85.7% (150)	90.9% (159)	74.0% (131)	
Sometimes	21.6% (44)	19.1% (27)	27.0% (17)		13.1% (23)	6.3% (11)	20.3% (36)	
Often	5.4% (11)	4.3% (6)	7.9% (5)		1.1% (2)	2.9% (5)	5.6% (10)	
*At disadvantage because of CHD*				< 0.001				< 0.001
Never	66.5% (135)	76.4% (107)	44.4% (28)		91.5% (161)	86.3% (151)	67.6% (119)	
Sometimes	31.0% (63)	21.4% (30)	52.4% (33)		6.8% (12)	12.0% (21)	30.7% (54)	
Often	2.5% (5)	2.1% (3)	3.2% (2)		1.7% (3)	1.7% (3)	1.7% (3)	

^*^p-value calculated on the differences between 2011 and 2021 on the 177 consecutive patients

^**^Psychologist, psychiatrist or RIAGG “Regionale Instelling voor Ambulante Geestelijke Gezondheidszorg” (Regional Institution for Oautpatient Mental Health Care)

In 2021, significantly less patients had contact with other CHD patients (*p* = 0.026) when compared to the previous follow-up.

### Psychosocial functioning

**Fig. 1:**
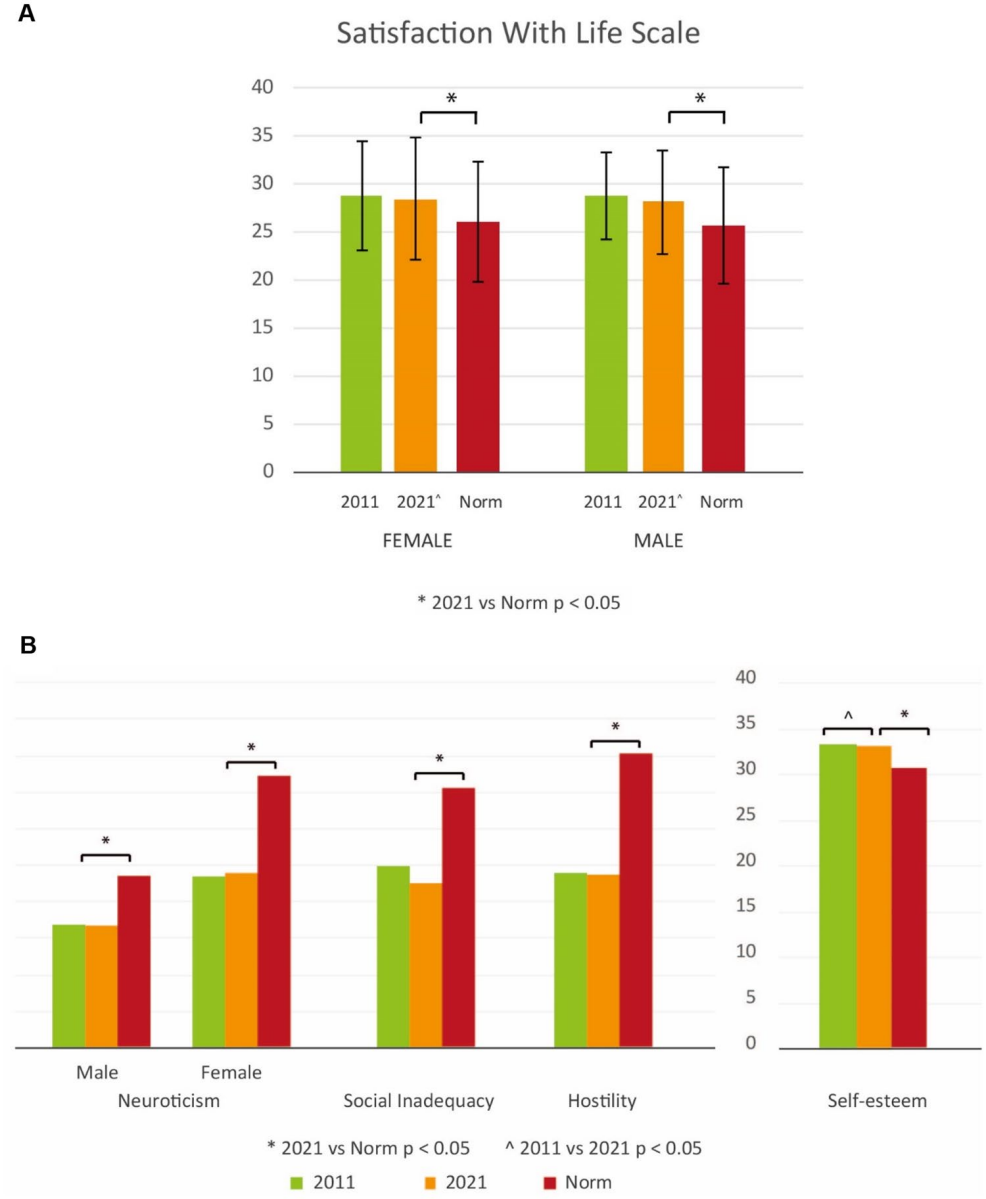
**A** Satisfaction with Life and overall quality of life of patients with CHD in 2011 and 2021 compared with normative data per gender. **B** Dutch personality Questionnaire of patients with CHD in 2011, 2021 compared with normative data. A high score is less favorable on neuroticism, hostility and social inadequacy. On self-esteem, a higher score is favorable

Patients with CHD were more satisfied with their life and experienced a higher quality of life than the norm (*p* < 0.001) (Fig. 1A). No significant difference was found between CHD diagnostic groups and over time. (Table 1S Supplementary material). Results of the prediction analysis are reported in Table 3S (Supplementary material).

With regard to psychosocial functioning of male and female patients, adults with CHD showed significantly lower (better) scores than the same gender peers from general population in terms of neuroticism (both females and males), social inadequacy and hostility (*p* < 0.001 for all subscales) (Fig. 1B). In addition, the CHD group showed a significantly higher self-esteem (*p* < 0.001) than the norm. No difference was found between mild and moderate/severe CHD. However, compared to 10 years ago, our cohort reported significantly lower self-esteem (*p* = 0.025). Results of the prediction analysis are reported in Tables 4S, 5S, 6S, 7S (Supplementary material).

Regarding perceived social support, our cohort obtained on average 86% of the total maximum score for social support from family, friends and a special person. No norm data are available. No significant difference was found overtime and between mild and moderate/severe CHD in term of perceived social support. (Table 1S, Supplementary material).

## Discussion

Overall, 40–53 years after surgery, adults with CHD achieved a lower educational and occupation level and lower employment rate when compared to the normative data. In comparison with 10 years ago, a more pessimistic view regarding the outcomes of their CHD was reported. Nonetheless, the quality of life of our cohort was higher than the general Dutch population.

A lower education level was also found in a recent systematic review which reported that patients with CHD achieved less often secondary or higher education or vocational training when compared to their peers [24]. Patients with CHD have been at higher risk of neurodevelopmental impairment since their childhood, which could be related to both the CHD itself and use of cardiopulmonary bypass during the surgery [25]. However, the precise impact on their educational achievement is yet unknown. In addition, children with CHD may have a reduced school attendance due to frequent cardiological outpatient visit and/or higher chance of longer school absenteeism due to repeated re-hospitalization for arrhythmia’s, infections and re-operations [26]. Furthermore, parents may demand less in terms of education and achievements from their CHD children because of their medical history. In concert with a lower educational level in our patient sample, adults with CHD also had a lower occupation level and employment rate as compared to the general Dutch population. However, no significant changes in educational, occupational level and employment rate were found over time. In contrast with the majority of previous studies, we did not find a significant difference between CHD diagnoses in terms of employment rate [27–29]. This could be related to the different selection of CHD per study. For instance, patients with more severe conditions such as Fontan circulation or more complex congenital heart diseases were not included in our study. This may explain the non-significant difference in employment rate between mild and moderate/severe CHD.

Another interesting finding was the increased number of patients unable to work since the last follow-up in 2011. In fact, in 2011, 1,1% was unable to work (0.5% of the total sample was disabled), whereas in 2021, 7.9% of our cohort was unable to work (disabled or on long-term sick leave). Moreover, significantly more patients considered their CHD to be severe during the last follow-up time and more experienced lack of physical strength and felt physically restricted and disadvantaged.  A possible explanation to these results may be related to the Covid-19 pandemic. Patients with CHD may felt more at risk of possible complications than the general population because of their cardiac condition. In fact, the Covid-19 stringency index reflects only the governmental policies, but we have no further information regarding the infection status of our cohort and neither how the pandemic affected the lives of our patients [22, 23].

These factors may have led them to consider their congenital condition as more severe at this follow-up time. Consequently, the fact that patients with more severe diagnosis felt more restricted because of their CHD can also be explained by the major physical burden of their condition and older age [30]. In addition, significantly lower scores in term of self-esteem were found in this follow-up time in comparison with 2011, even though they remained significantly higher than the normal population [2]. Generally, a peak of self-esteem is reported at the age of 50 or 60 with a decline at older age. However, a decrease in self-esteem is mostly related to 2 reasons: loss of social position or reduction in physical ability [31, 32]. Even though the exercise capacity of our cohort was comparable with that of a healthy person of same age and gender and higher than in 2011, patients who scored better at the exercise test reported better self-esteem.

Nonetheless, adults with CHD obtained better scores on quality of life, emotional and social functioning when compared to normative data. These findings were comparable with the previous follow-ups and with the literature [3, 4, 8, 17, 33, 34]. In contrast with the American literature which showed significant higher prevalence rates of psychological symptoms in patients with CHD, European studies suggested more favorable outcomes [35–38]. In fact, our previous studies showed comparable levels of psychopathology in CHD patients and the norm population [7, 14]. Moreover, patients with CHD scored significantly better in terms of neuroticism, social inadequacy and hostility when compared to the norm. Even if these findings seem to be in contradiction with a more pessimistic view of our patients on their CHD, it has to be considered that our cohort of patients went through a life-threatening experience in their childhood, which may have resulted in a stronger sense of coherence (SOC). According to Antonovsky’s theory, SOC defines the ability to cope with everyday stressors and it consists of comprehensibility, manageability and meaningfulness. The exposure to stressful life events during life and successful management of them help to strengthen their SOC [39]. Furthermore, in our study, we found that patients with higher satisfaction with life had a better exercise capacity, were more often married and had a higher occupational level. These factors have been shown to be linked to stronger sense of coherence (SOC), which is also associated with better quality of life in CHD patients [40].

## Strengths and limitations

In this unique longitudinal study, psychosocial outcomes of a cohort of patients with CHD were investigated every 10 years up to now 40–53 years after surgery. Using the same assessment instruments, we assessed psychosocial outcome every 10 years (2001, 2011 and 2021) after surgery.

This study included a consecutive series of operated patients with 5 different CHD diagnosis (ASD, VSD, PS, ToF and TGA). Therefore, the results obtained may not be generalizable to other CHD not included in the study.

Furthermore, normative data were not available for all biographical characteristics, which can make the interpretation of the results more complex.

This study was conducted during the Covid-19 pandemic which could have influenced the psychosocial outcomes of our cohort.

## Conclusions

Forty to 53 years after surgery, CHD adults reported a better quality of life and psychological functioning when compared to the normative data, despite the fact they had a lower educational and occupational level, and lower employment rate. Over time, the biographical characteristics normalized and it can be concluded that these adults are generally able to live a normal life. Counseling and support for CHD patients in choosing an education and occupation path should already be considered in their adolescence.

At this follow-up time, patients with CHD, especially patients with a more complex  diagnosis, experienced their CHD as more severe; they perceived more lack of physical strength and felt more often physically restricted and at disadvantage compared to 10 and 20 years ago. Therefore, attention for the self-perceived medical condition is warranted. Furthermore, when possible, reassuring patients regarding their CHD could be a fundamental intervention to improve the mental well-being of these adults.

## Supplementary Information

Below is the link to the electronic supplementary material.

Supplementary file1 (DOCX 54 KB)

## Data Availability

The anonymous data that support the findings of this study are available from the author, upon reasonable request.
