# Supplementary material for: Daily life and psychosocial functioning of adults with congenital heart disease: a 40–53 years after surgery follow-up study
Source: Clin Res Cardiol. 2022 Dec 19;112(7):880–90. doi: 10.1007/s00392-022-02132-w (PMC9761041; doi:10.1007/s00392-022-02132-w)
Supplement: Supplementary file 1 — Supplementary file1 (DOCX 54 KB) [file 392_2022_2132_MOESM1_ESM.docx]

**Supplementary material:**

**Table 1S.** Differences between CHD diagnosis in the quality of life questionnaires: Satisfaction with life scale (SWLS), Perceived social support(PSSS) and Dutch personality questionnaire(DPQ)

| **CHD classification** | | | |
| --- | --- | --- | --- |
|  | **Mild CHD** | **Moderate/severe CHD** | **p** |
| SWLS | 28.3 ± 5.8 | 28.1 ± 5.8 | 0.695 |
| PSSS12 | 71.7 ± 11.1 | 73.4 ± 10.3 | 0.213 |
| NPV |  |  |  |
| Neuroticism | 8.06 ± 7.88 | 8.08 ± 7.45 | 0.985 |
| Social inadequacy | 8.9 ± 7.5 | 9.3 ± 9.2 | 0.760 |
| Hostility | 9.4 ± 7.6 | 9.7 ± 8.2 | 0.853 |
| Self-esteem | 33.7 ± 6.2 | 32.1 ± 6.2 | 0.088 |
| SWLS= Satisfaction with life scale, PSSS12=Perceived Social Support Scale, DPQ=Dutch Personality Questionnaire | | | |
|  | | | |

Lower scores define better outcomes on the neuroticism, social inadequacy and hostility scale. On the self-esteem, SWLS and PSSS12 scales, higher scores define better outcomes.

**Table 2S.** The Dutch personality Questionnaire (DPQ) of patients with CHD in 2011, 2021 compared with normative data.

|  | **Mean scores on the DPQ** | | | | |
| --- | --- | --- | --- | --- | --- |
|  | **2011** | **2021** | **Norm** | **p 2021 vs 2011** | **p 2021 vs norm** |
| Neuroticism |  |  |  |  |  |
| Female | 9.4 ± 8.0 | 9.6 ± 8.4 | 14.8 ± 9.9 | 0.116 | <0.001 |
| Male | 6.8 ± 6.1 | 6.7 ± 6.9 | 9.3 ± 8.9 | 0.706 | <0.001 |
| Social Inadequacy | 10 ± 7.7 | 9.0 ± 8.0 | 14.1 ± 9.8 | 0.864 | <0.001 |
| Hostility | 9.6 ± 7.4 | 9.5 ± 7.8 | 16 ± 8.1 | 0.629 | <0.001 |
| Self-esteem | 33.3 ± 6.5 | 33.2 ± 6.2 | 30.5 ± 7 | 0.025 | <0.001 |

Lower scores define better outcomes on the neuroticism, social inadequacy and hostility. On the self-esteem scale, higher scores define better outcomes.

**Table3S.** Prediction of outcome for Satisfaction with life scale

|  | **Satisfaction with Life Scale** | | | | | |
| --- | --- | --- | --- | --- | --- | --- |
|  | **Univariable** | | **Multivariable** | | | |
|  | **β** | **p** | **β** | **B** | **95% CI** | **p** |
| Age (years) | -0.032 | 0.653 | - | - | - | - |
| Gender (reference category: male) | 0.023 | 0.744 | - | - | - | - |
| Cardiac medications | -0.329 | <0.001 | - | - | - | - |
| Exercise capacity | 0.310 | <0.001 | 0.273 | 0.067 | [0.029; 0.105] | <0.001 |
| Systemic function (reference category: good) |  |  |  |  |  |  |
| Reasonable | 0.043 | 0.565 | - | - | - | - |
| Moderate | -0.210 | 0.006 | -0.206 | -3.781 | [-6.538;-1.024] | 0.008 |
| Bad | -0.105 | 0.157 | - | - | - | - |
| Cardiac diagnosis (reference category: ASD) |  |  |  |  |  |  |
| VSD | 0.149 | 0.070 | - | - | - | - |
| PS | 0.156 | 0.047 | - | - | - | - |
| ToF | 0.144 | 0.077 | - | - | - | - |
| TGA | -0.058 | 0.445 | - | - | - | - |
| Covid-19 Stringency index | -0.071 | 0.318 | - | - | - | - |
| Living conditions (reference category: independently) |  |  |  |  |  |  |
| With parents | -0.063 | 0.372 | - | - | - | - |
| Institution | -0.029 | 0.681 | - | - | - | - |
| Other | 0.100 | 0.157 | 0.211 | 9.853 | [2.915;16.790] | 0.006 |
| Education level (reference category: elementary) : |  |  |  |  |  |  |
| Average | 0.185 | 0.022 | - | - | - | - |
| High | 0.185 | 0.022 | - | - | - | - |
| Occupation level (reference category: elementary) |  |  |  |  |  |  |
| Lower | 0.464 | <0.001 | 0.324 | 3.664 | [0.586;6.743] | 0.020 |
| Average | 0.493 | <0.001 | 0.309 | 3.805 | [0.593; 7.018] | 0.021 |
| Scientific | 0.486 | <0.001 | 0.264 | 3.112 | [-0.010;6.235] | 0.051 |
|  |  |  |  |  |  |  |
| Marital status (reference category: married): |  |  |  |  |  |  |
| (No) stable relationship | -0.206 | 0.003 | -0.168 | -2.093 | [-3.950;-0.235] | 0.028 |
| Divorced | -0.153 | 0.027 | -0.191 | -3.304 | [-5.866; -0.741] | 0.012 |
| Widowed | -0.264 | <0.001 | -0.178 | -8.319 | [-15.188; -1.449] | 0.018 |

95%CI= 95% confident interval; ASD: Atrial Septal Defect, VSD: ventricular septal defect, PS: Pulmonary Stenosis, ToF: Tetralogy of Fallot, TGA: Transposition of the Great Arteries

*Covid-19 Stringency index [1, 2].

**Table 4S.** Prediction of outcome for Neuroticism (DPQ)

|  | **DPQ- NEUROTICISM** | | | | | |
| --- | --- | --- | --- | --- | --- | --- |
|  | **Univariate** | | **Multivariate** | | | |
|  | **β** | **p** | **β** | **B** | **95% CI** | **p** |
| Age (years) | -0.046 | 0.512 | - | - | - | - |
| Gender (reference category: male) | 0.188 | 0.007 | 0.229 | 3.136 | [0.979; 5.294] | 0.005 |
| Cardiac medications | 0.247 | <0.001 | 0.161 | 2.367 | [-0.009;4.743] | 0.051 |
| Exercise capacity | -0.327 | <0.001 | -0.283 | -0.086 | [-0.135;-0.037] | <0.001 |
| Systemic function (reference category: good) |  |  |  |  |  |  |
| Reasonable | -0.070 | 0.361 | - | - | - | - |
| Moderate | 0.110 | 0.152 | - | - | - | - |
| Bad | 0.036 | 0.635 | - | - | - | - |
| Cardiac diagnosis (reference category: ASD) |  |  |  |  |  |  |
| VSD | -0.007 | 0.934 | - | - | - | - |
| PS | -0.043 | 0.591 | - | - | - | - |
| ToF | -0.072 | 0.383 | - | - | - | - |
| TGA | 0.088 | 0.260 | - | - | - | - |
| Covid-19 Stringency index | 0.096 | 0.172 | - | - | - | - |
| Living conditions (reference category: independently) |  |  | - | - | - | - |
| With parents | 0.007 | 0.923 | - | - | - | - |
| Institution | -0.015 | 0.829 | - | - | - | - |
| Other | 0.135 | 0.056 | - | - | - | - |
| Education level (reference category: elementary) : |  |  | - | - | - | - |
| Average | -0.241 | 0.003 | - | - | - | - |
| High | -0.266 | <0.001 | - | - | - | - |
| Occupation level (reference category: elementary) |  |  | - | - | - | - |
| Lower | -0.253 | 0.057 | - | - | - | - |
| Average | -0.311 | 0.014 | - | - | - | - |
| Scientific | -0.414 | 0.001 | - | - | - | - |
|  |  |  | - | - | - | - |
| Marital status (reference category: married): |  |  | - | - | - | - |
| (No) stable relationship | 0.080 | 0.270 | - | - | - | - |
| Divorced | 0.154 | 0.034 | 0.169 | 3.668 | [0.369;6.966] | 0.030 |
| Widowed | 0.041 | 0.559 | - | - | - | - |

95%CI= 95% confident interval; ASD: Atrial Septal Defect, VSD: ventricular septal defect, PS: Pulmonary Stenosis, ToF: Tetralogy of Fallot, TGA: Transposition of the Great Arteries

*Covid-19 Stringency index [1, 2].

**Table 5S.** Prediction of outcome for Social Inadequacy (DPQ)

|  | **DPQ- SOCIAL INADEQUANCY** | | | | | |
| --- | --- | --- | --- | --- | --- | --- |
|  | **Univariate** | | **Multivariate** | | | |
|  | **β** | **p** | **β** | **B** | **95% CI** | **p** |
| Age (years) | -0.144 | 0.041 | -0.168 | -0.277 | [-0.546; -0.008] | 0.044 |
| Gender (reference category: male) | 0.044 | 0.535 | - | - | - | - |
| Cardiac medications | 0.098 | 0.185 | - | - | - | - |
| Exercise capacity | -0.170 | 0.022 | -0.150 | -0.055 | [-0.114;0.004] | 0.069 |
| Systemic function (reference category: good) |  |  | - | - | - | - |
| Reasonable | 0.002 | 0.982 | - | - | - | - |
| Moderate | 0.053 | 0.493 | - | - | - | - |
| Bad | -0.012 | 0.872 | - | - | - | - |
| Cardiac diagnosis (reference category: ASD) |  |  | - | - | - | - |
| VSD | 0.007 | 0.937 | - | - | - | - |
| PS | 0.086 | 0.280 | - | - | - | - |
| ToF | 0.040 | 0.626 | - | - | - | - |
| TGA | 0.031 | 0.689 | - | - | - | - |
| Covid-19 Stringency index | 0.023 | 0.745 | - | - | - | - |
| Living conditions (reference category: independently) |  |  | - | - | - | - |
| With parents | -0.020 | 0.773 | - | - | - | - |
| Institution | -0.045 | 0.503 | - | - | - | - |
| Other | -0.048 | 0.526 | - | - | - | - |
| Education level (reference category: elementary) : |  |  | - | - | - | - |
| Average | -0.139 | 0.086 | - | - | - | - |
| High | -0.158 | 0.051 | - | - | - | - |
| Occupation level (reference category: elementary) |  |  | - | - | - | - |
| Lower | -0.117 | 0.388 | - | - | - | - |
| Average | -0.217 | 0.090 | - | - | - | - |
| Scientific | -0.087 | 0.506 | - | - | - | - |
|  |  |  | - | - | - | - |
| Marital status (reference category: married): |  |  | - | - | - | - |
| (No) stable relationship | -0.140 | 0.054 | -0.169 | -3.094 | [-6.018;-0.169] | 0.038 |
| Divorced | -0.001 | 0.986 | - | - | - | - |
| Widowed | -0.84 | 0.235 | - | - | - | - |

95%CI= 95% confident interval; ASD: Atrial Septal Defect, VSD: ventricular septal defect, PS: Pulmonary Stenosis, ToF: Tetralogy of Fallot, TGA: Transposition of the Great Arteries

*Covid-19 Stringency index [1, 2].

**Table 6S.** Prediction of outcome for hostility (DPQ)

|  | **DPQ- HOSTILITY** | | | | | |
| --- | --- | --- | --- | --- | --- | --- |
|  | **Univariate** | | **Multivariate** | | | |
|  | **β** | **p** | **β** | **B** | **95% CI** | **p** |
| Age (years) | -0.084 | 0.237 | - | - | - | - |
| Gender (reference category: male) | -0.055 | 0.439 | - | - | - | - |
| Cardiac medications | 0.117 | 0.111 | - | - | - | - |
| Exercise capacity | -0.250 | <0.001 | -0.276 | -0.094 | [-0.147;-0.041] | <0.001 |
| Systemic function (reference category: good) |  |  | - | - | - | - |
| Reasonable | 0.001 | 0.990 | - | - | - | - |
| Moderate | 0.144 | 0.062 | - | - | - | - |
| Bad | -0.004 | 0.956 | - | - | - | - |
| Cardiac diagnosis (reference category: ASD) |  |  | - | - | - | - |
| VSD | 0.069 | 0.410 | - | - | - | - |
| PS | -0.29 | 0.712 | - | - | - | - |
| ToF | -0.026 | 0.751 | - | - | - | - |
| TGA | 0.109 | 0.160 | - | - | - | - |
| Covid-19 Stringency index | 0.023 | 0.749 | - | - | - | - |
| Living conditions (reference category: independently) |  |  | - | - | - | - |
| With parents | 0.014 | 0.841 | - | - | - | - |
| Institution | 0.035 | 0.616 | - | - | - | - |
| Other | 0.110 | 0.122 | - | - | - | - |
| Education level (reference category: elementary) : |  |  | - | - | - | - |
| Average | -0.104 | 0.191 | - | - | - | - |
| High | -0.256 | 0.001 | - | - | - | - |
| Occupation level (reference category: elementary) |  |  | - | - | - | - |
| Lower | -0.162 | 0.223 | - | - | - | - |
| Average | -0.267 | 0.035 | - | - | - | - |
| Scientific | -0.334 | 0.010 | - | - | - | - |
|  |  |  | - | - | - | - |
| Marital status (reference category: married): |  |  | - | - | - | - |
| (No) stable relationship | 0.040 | 0.581 | - | - | - | - |
| Divorced | 0.144 | 0.047 | 0.154 | 3.707 | [-0.060; 7.473] | 0.054 |
| Widowed | -0.058 | 0.413 | - | - | - | - |

95%CI= 95% confident interval; ASD: Atrial Septal Defect, VSD: ventricular septal defect, PS: Pulmonary Stenosis, ToF: Tetralogy of Fallot, TGA: Transposition of the Great Arteries

*Covid-19 Stringency index [1, 2].

**Table 7S.** Prediction of outcome for self-esteem (DPQ)

|  | **DPQ- SELF-ESTEEM** | | | | | |
| --- | --- | --- | --- | --- | --- | --- |
|  | **Univariate** | | **Multivariate** | | | |
|  | **β** | **p** | **β** | **B** | **95% CI** | **p** |
| Age (years) | 0.104 | 0.141 | - | - | - | - |
| Gender (reference category: male) | -0.135 | 0.055 | - | - | - | - |
| Cardiac medications | -0.244 | <0.001 | - | - | - | - |
| Exercise capacity | 0.327 | <0.001 | 0.287 | 0.075 | [0.035;0.114] | <0.001 |
| Systemic function (reference category: good) |  |  | - | - | - | - |
| Reasonable | -0.025 | 0.744 | - | - | - | - |
| Moderate | -0.146 | 0.058 | - | - | - | - |
| Bad | -0.087 | 0.249 | - | - | - | - |
| Cardiac diagnosis (reference category: ASD) |  |  | - | - | - | - |
| VSD | 0.031 | 0.711 | - | - | - | - |
| PS | 0.068 | 0.394 | - | - | - | - |
| ToF | -0.051 | 0.534 | - | - | - | - |
| TGA | -0.103 | 0.185 | - | - | - | - |
| Covid-19 Stringency index | -0.096 | 0.172 | - | - | - | - |
| Living conditions (reference category: independently) |  |  | - | - | - | - |
| With parents | -0.116 | 0.102 | -0.250 | -17.454 | [-28.003;-6.904] | 0.001 |
| Institution | -0.044 | 0.535 | - | - | - | - |
| Other | 0.067 | 0.343 | - | - | - | - |
| Education level (reference category: elementary) : |  |  | - | - | - | - |
| Average | 0.239 | 0.003 | - | - | - | - |
| High | 0.287 | <0.001 | - | - | - | - |
| Occupation level (reference category: elementary) |  |  | - | - | - | - |
| Lower | 0.474 | <0.001 | - | - | - | - |
| Average | 0.536 | <0.001 | - | - | - | - |
| Scientific | 0.561 | <0.001 | - | - | - | - |
|  |  |  | - | - | - | - |
| Marital status (reference category: married): |  |  | - | - | - | - |
| (No) stable relationship | -0.127 | 0.080 | - | - | - | - |
| Divorced | -0.116 | 0.109 | -0.150 | -2.769 | [-5.551; 0.014] | 0.051 |
| Widowed | 0.013 | 0.855 | - | - | - | - |

95%CI= 95% confident interval; ASD: Atrial Septal Defect, VSD: ventricular septal defect, PS: Pulmonary Stenosis, ToF: Tetralogy of Fallot, TGA: Transposition of the Great Arteries

*Covid-19 Stringency index [1, 2].

**References:**

1. Hannah Ritchie, E.M., Lucas Rodés-Guirao, Cameron Appel, Charlie Giattino, Esteban Ortiz-Ospina, Joe Hasell, Bobbie Macdonald, Diana Beltekian and Max Roser. *Coronavirus Pandemic (COVID-19)*. 2020; Available from: <https://ourworldindata.org/coronavirus>.

2. Hale, T., et al., *A global panel database of pandemic policies (Oxford COVID-19 Government Response Tracker).*
